# Supplementary figures and images for: SARS-CoV-2 variants with reduced infectivity and varied sensitivity to the BNT162b2 vaccine are developed during the course of infection
Source: PLoS Pathog. 2022 Jan 12;18(1):e1010242. doi: 10.1371/journal.ppat.1010242 (PMC8789181; doi:10.1371/journal.ppat.1010242)

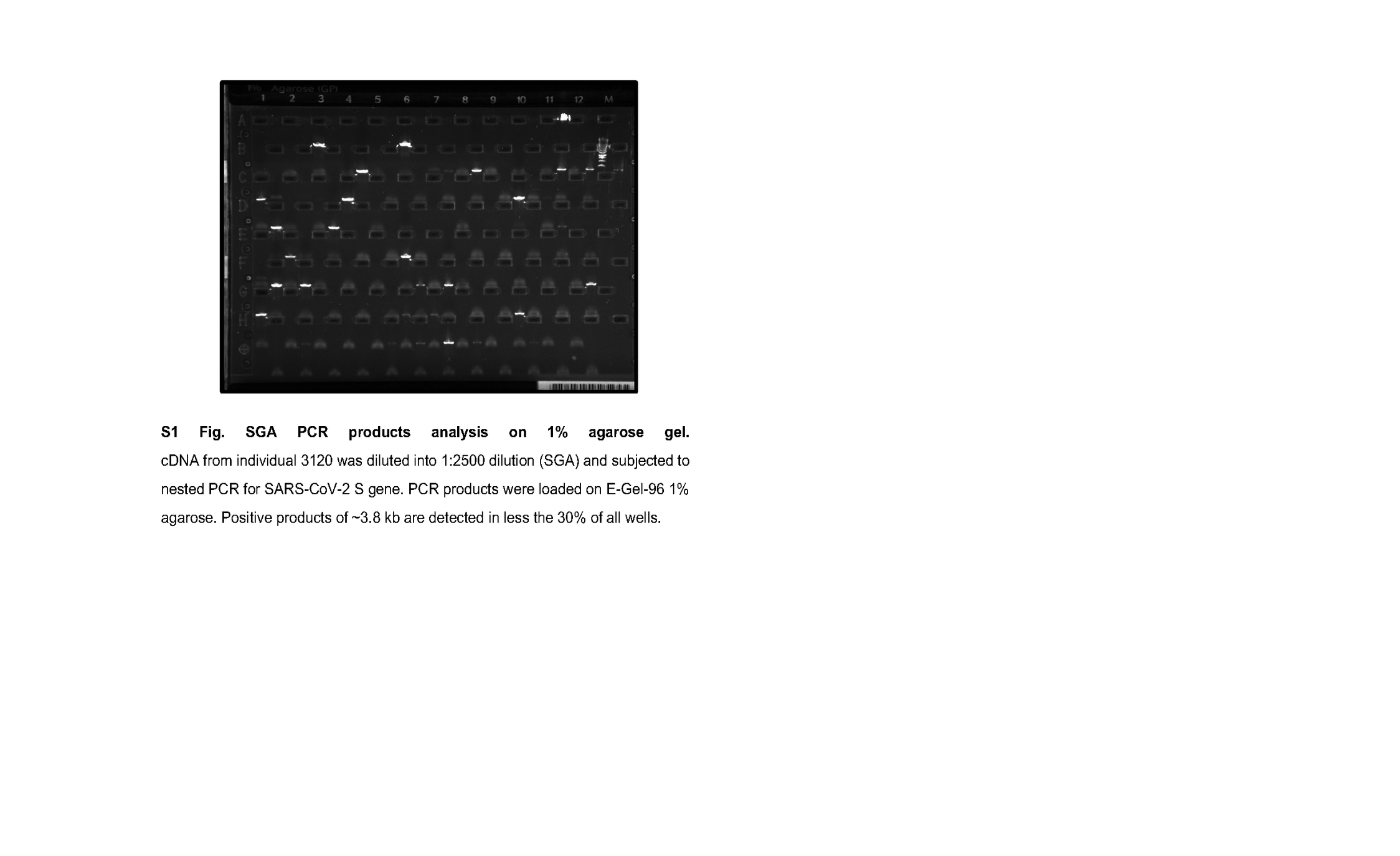

Supplement: S1 Fig — cDNA from individual 3120 was diluted into 1:2500 dilution (SGA) and subjected to nested PCR for SARS-CoV-2 S gene. PCR products were loaded on E-Gel-96 1% agarose. Positive products of ~3.8 kb are detected in less the 30% of all wells. (TIF) [file ppat.1010242.s001.tif]

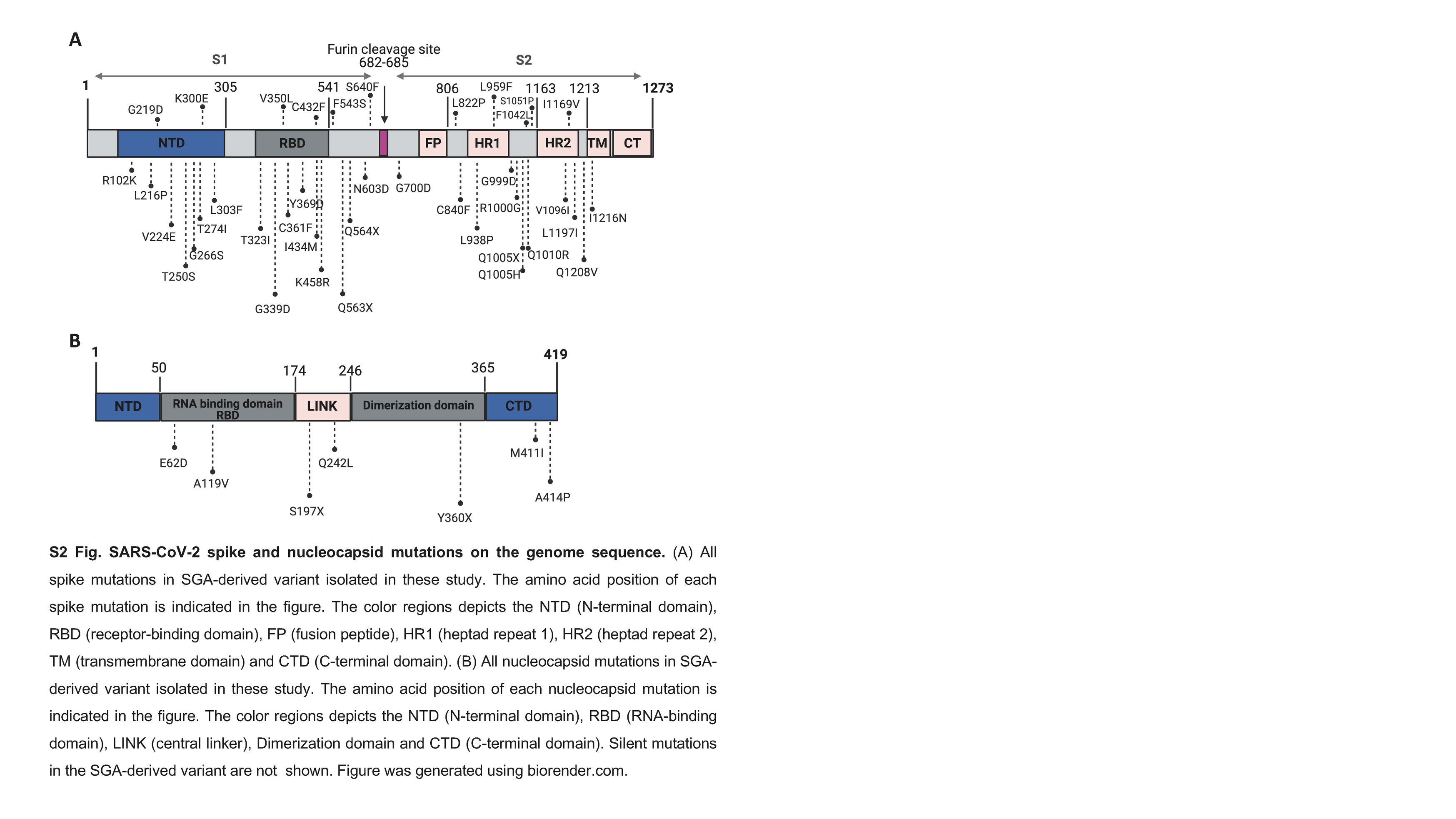

Supplement: S2 Fig — (A) All spike mutations in SGA-derived variant isolated in these study. The amino acid position of each spike mutation is indicated in the figure. The color regions depicts the NTD (N-terminal domain), RBD (receptor-binding domain), FP (fusion peptide), HR1 (heptad repeat 1), HR2 (heptad repeat 2), TM (transmembrane domain) and CTD (C-terminal domain). (B) All nucleocapsid mutations in SGA-derived variant isolated in these study. The amino acid position of each nucleocapsid mutation is indicated in the figure. The color regions depicts the NTD (N-terminal domain), RBD (RNA-binding domain), LINK (central linker), Dimerization domain and CTD (C-terminal domain). Silent mutations in the SGA-derived variant are not shown. (TIF) [file ppat.1010242.s002.tif]

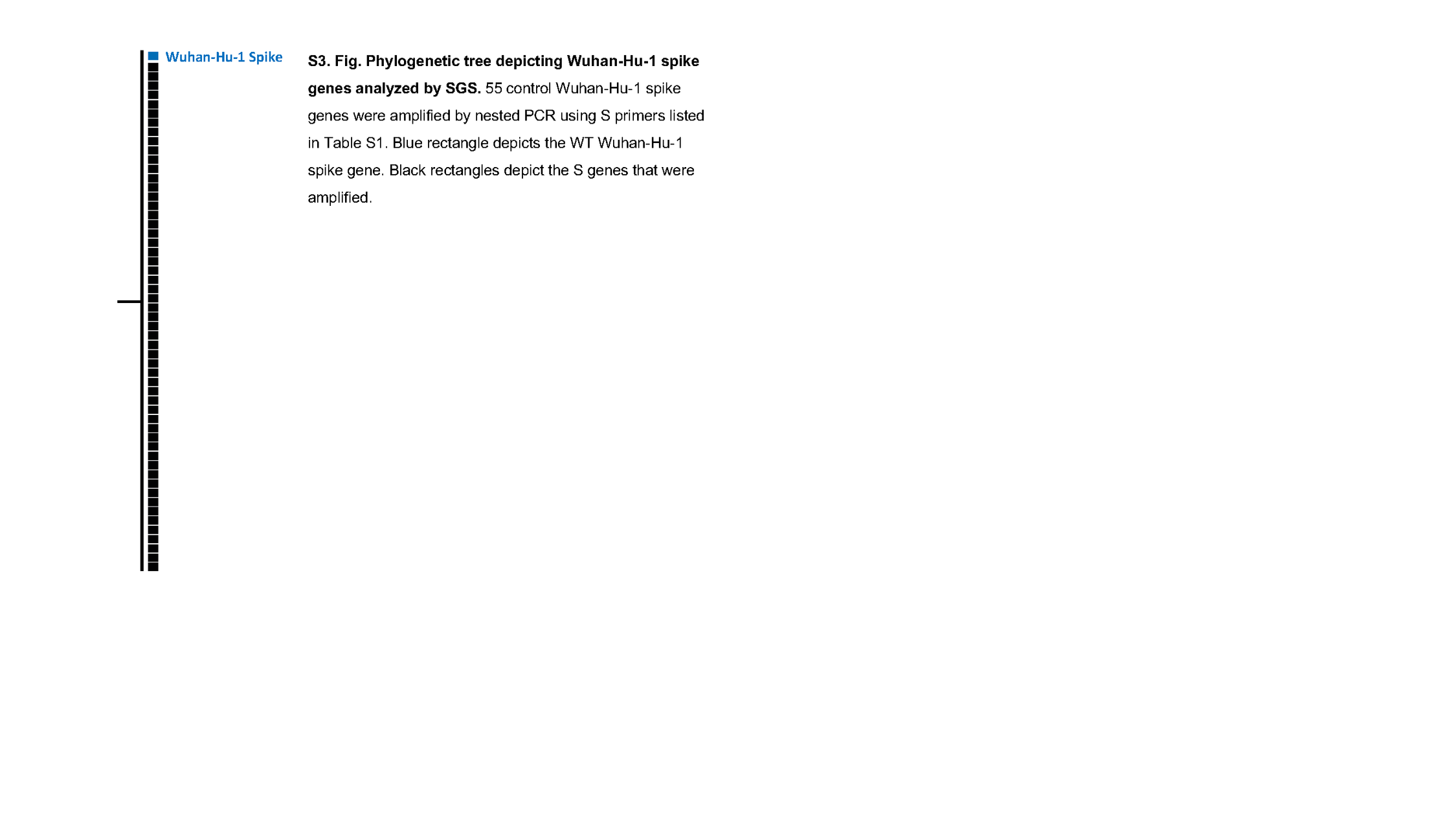

Supplement: S3 Fig — 55 control Wuhan-Hu-1 spike genes were amplified by nested PCR using S primers listed in S1 Table. Blue rectangle depicts the WT Wuhan-Hu-1 spike gene. Black rectangles depict the S genes that were amplified. (TIF) [file ppat.1010242.s003.tif]

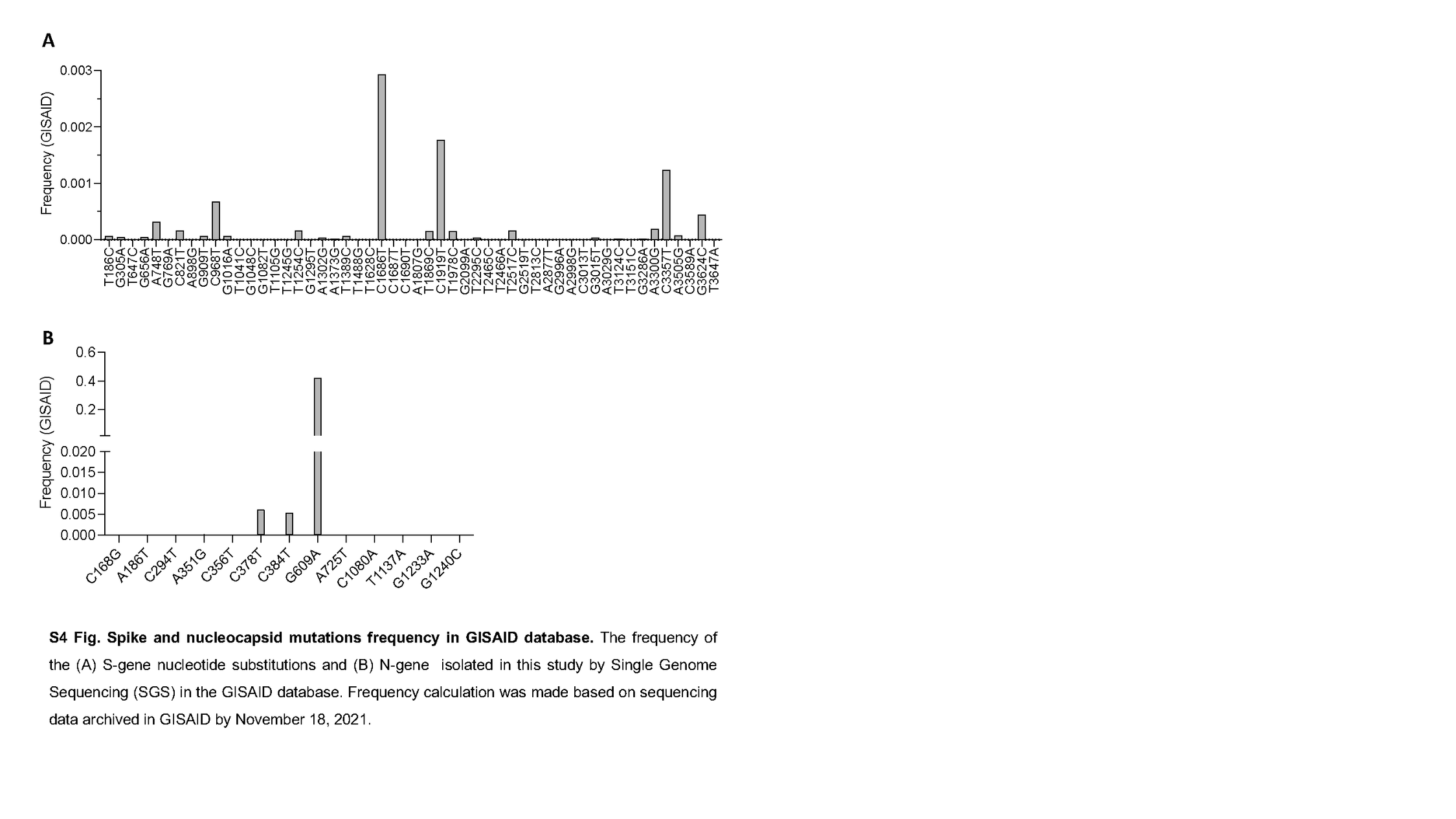

Supplement: S4 Fig — The frequency of the (A) S-gene nucleotide substitutions and (B) N-gene isolated in this study by Single Genome Sequencing (SGS) in the GISAID database. Frequency calculation was made based on sequencing data archived in GISAID by November 18, 2021. (TIF) [file ppat.1010242.s004.tif]

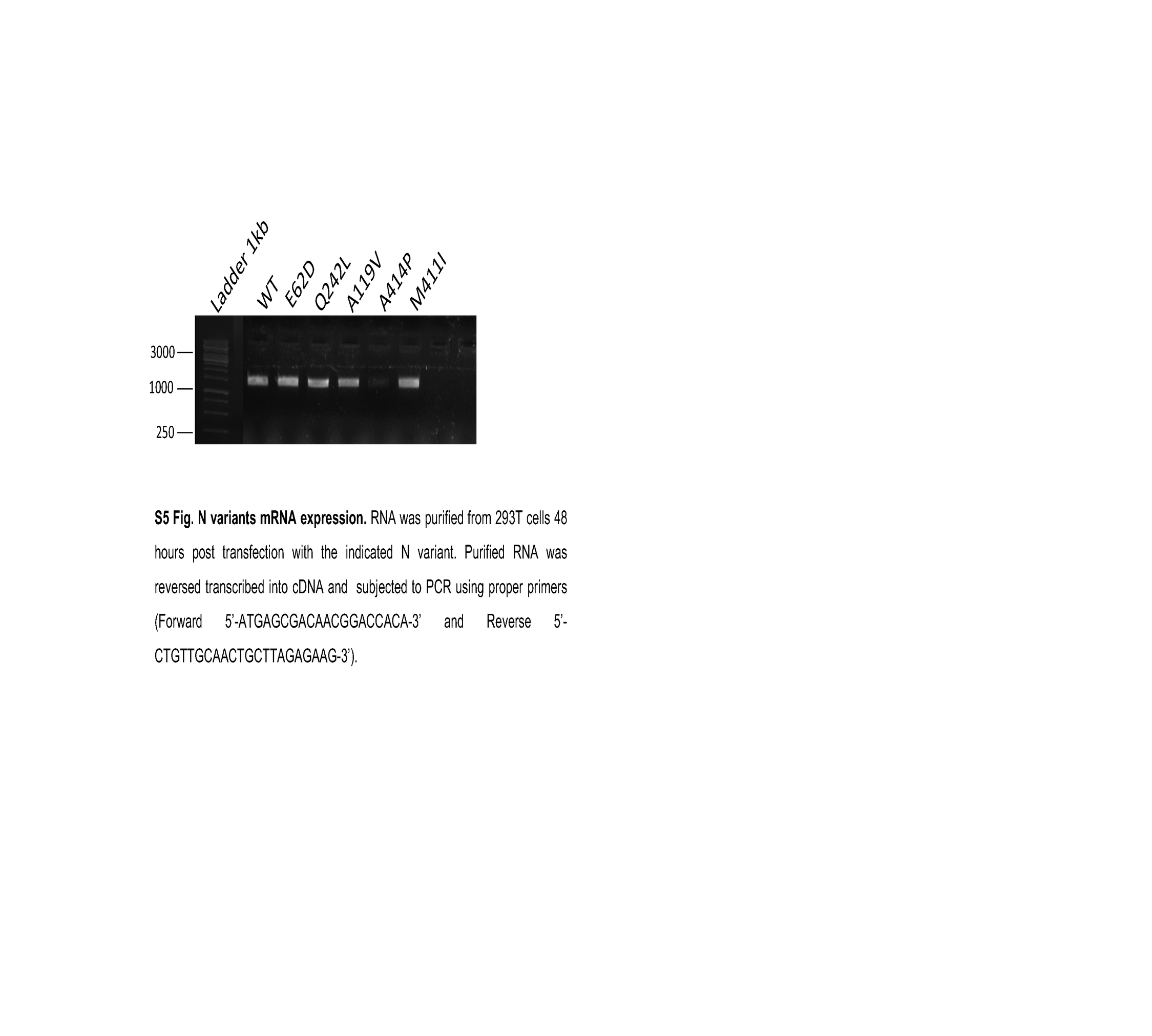

Supplement: S5 Fig — RNA was purified from 293T cells 48 hours post transfection with the indicated N variant. Purified RNA was reversed transcribed into cDNA and subjected to PCR using proper primers (Forward 5’-ATGAGCGACAACGGACCACA-3’ and Reverse 5’-CTGTTGCAACTGCTTAGAGAAG-3’). (TIF) [file ppat.1010242.s005.tif]

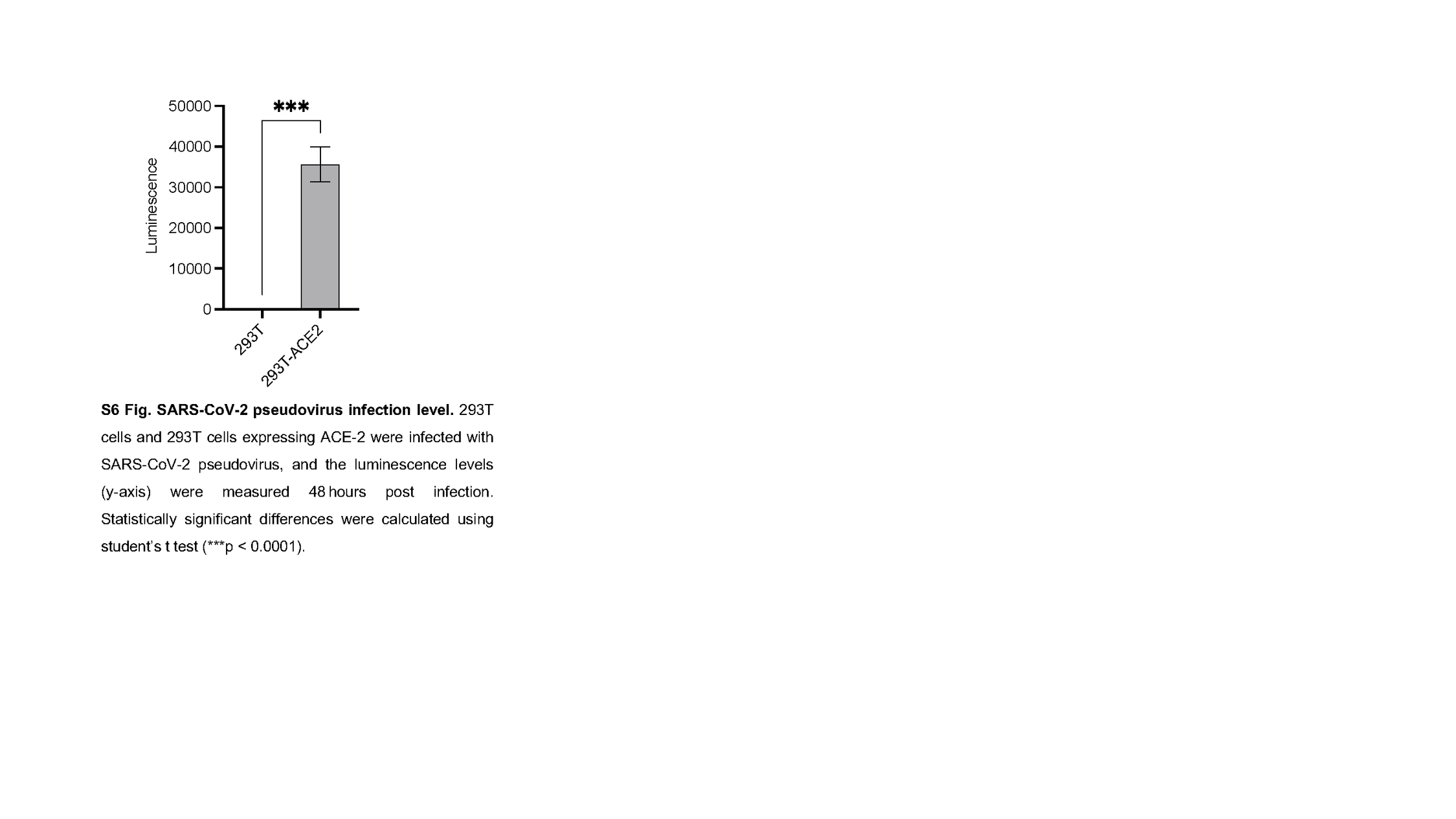

Supplement: S6 Fig — 293T cells and 293T cells expressing ACE-2 were infected with SARS-CoV-2 pseudovirus, and the luminescence levels (y-axis) were measured 48 hours post infection. Statistically significant differences were calculated using student’s t test (***p < 0.0001). (TIF) [file ppat.1010242.s006.tif]

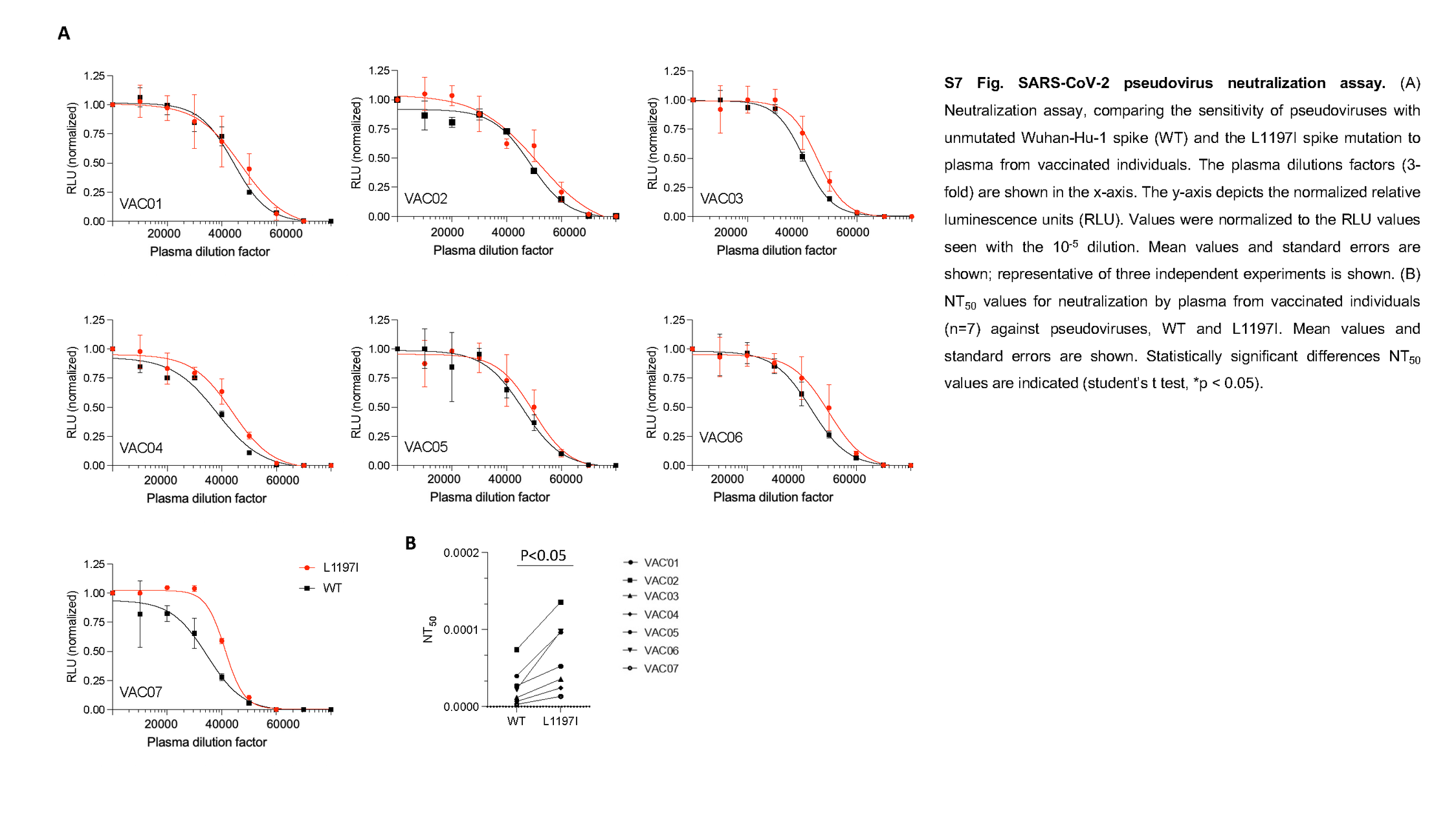

Supplement: S7 Fig — (A) Neutralization assay, comparing the sensitivity of pseudoviruses with unmutated Wuhan-Hu-1 spike (WT) and the L1197I spike mutation to plasma from vaccinated individuals. The plasma dilutions factors (3-fold) are shown in the x-axis. The y-axis depicts the normalized relative luminescence units (RLU). Values were normalized to the RLU values seen with the 10−5 dilution. Mean values and standard errors are shown; representative of three independent experiments is shown. (B) NT50 values for neutralization by plasma from vaccinated individuals (n = 7) against pseudoviruses, WT and L1197I. Mean values and standard errors are shown. Statistically significant differences NT50 values are indicated (student’s t test, *p < 0.05). (TIF) [file ppat.1010242.s007.tif]

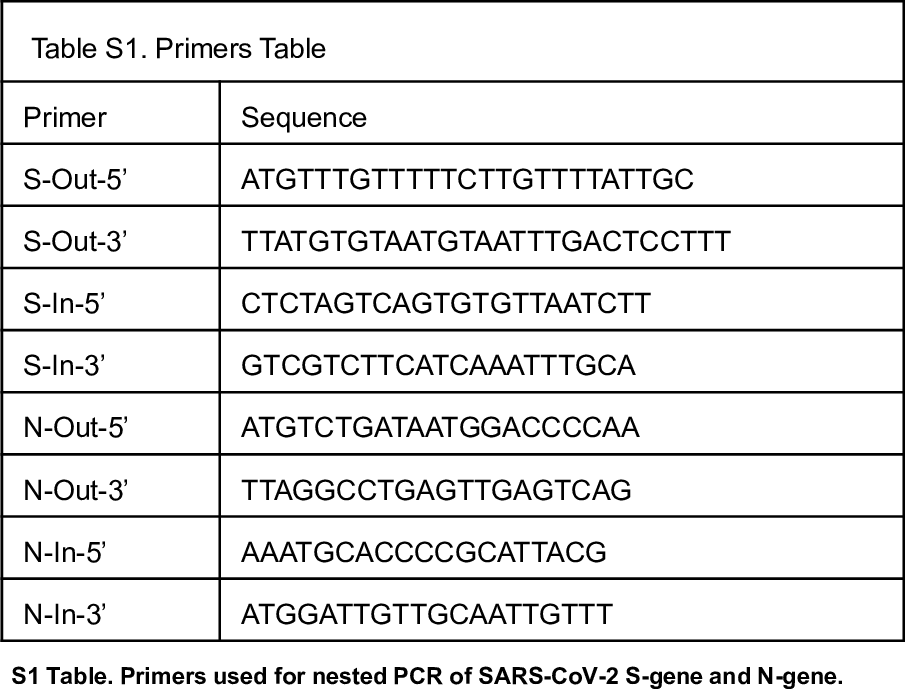

Supplement: S1 Table — (TIF) [file ppat.1010242.s008.tif]

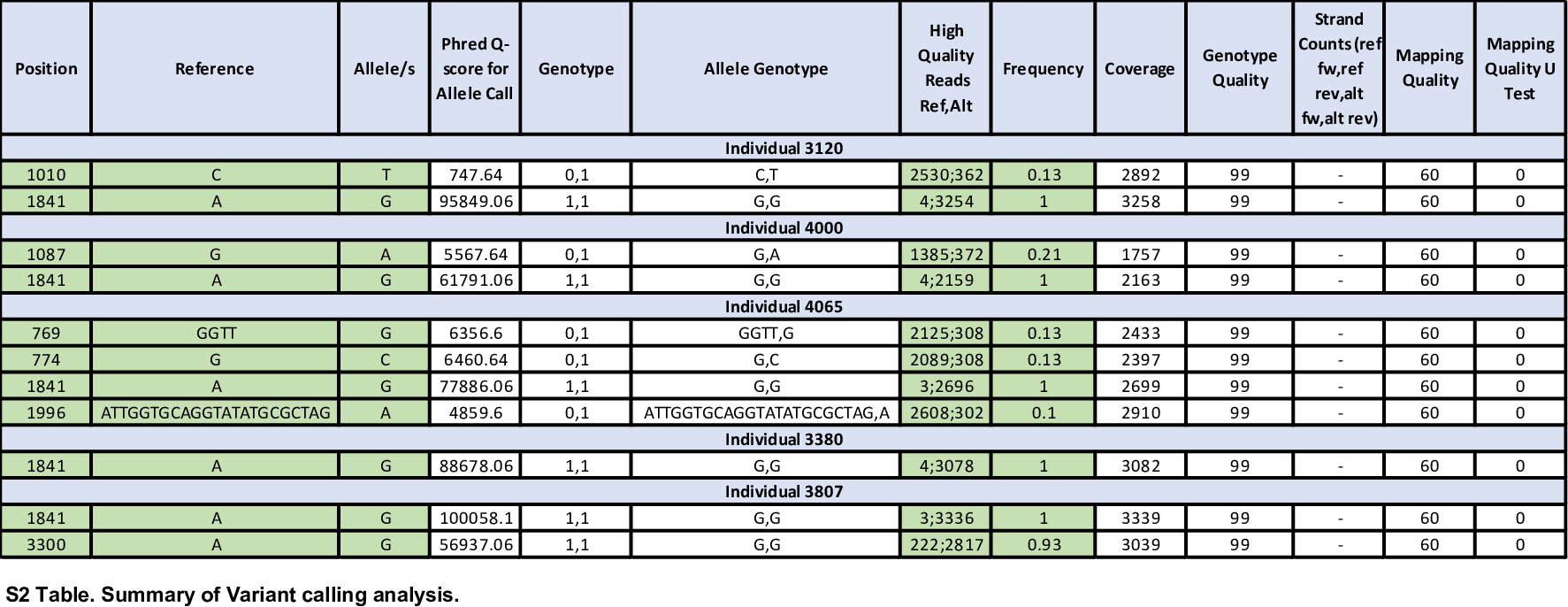

Supplement: S2 Table — (TIF) [file ppat.1010242.s009.tif]

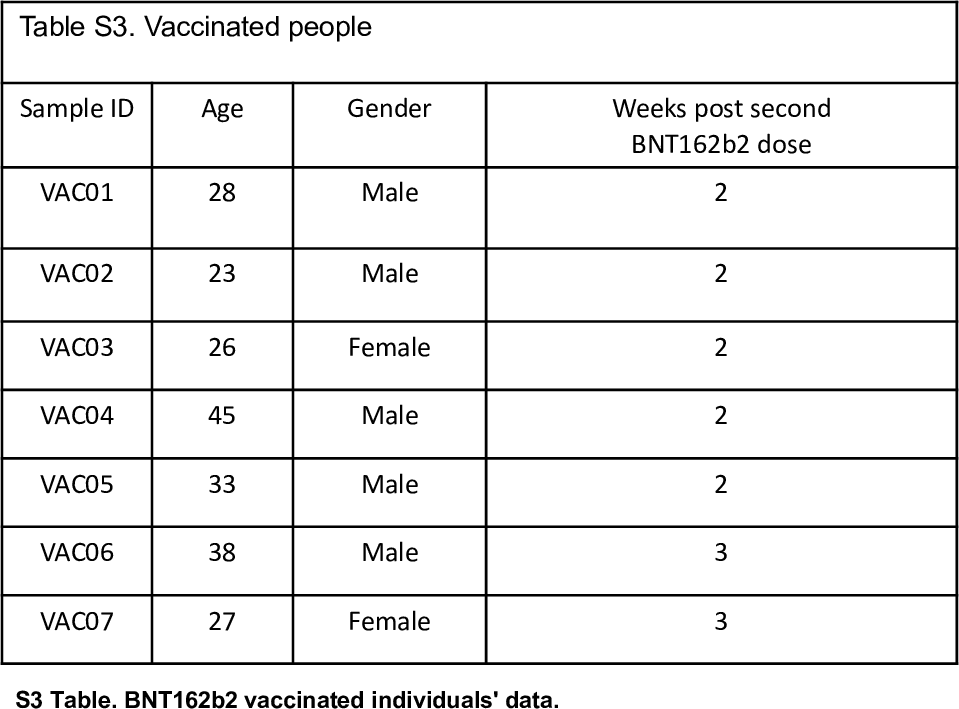

Supplement: S3 Table — (TIF) [file ppat.1010242.s010.tif]

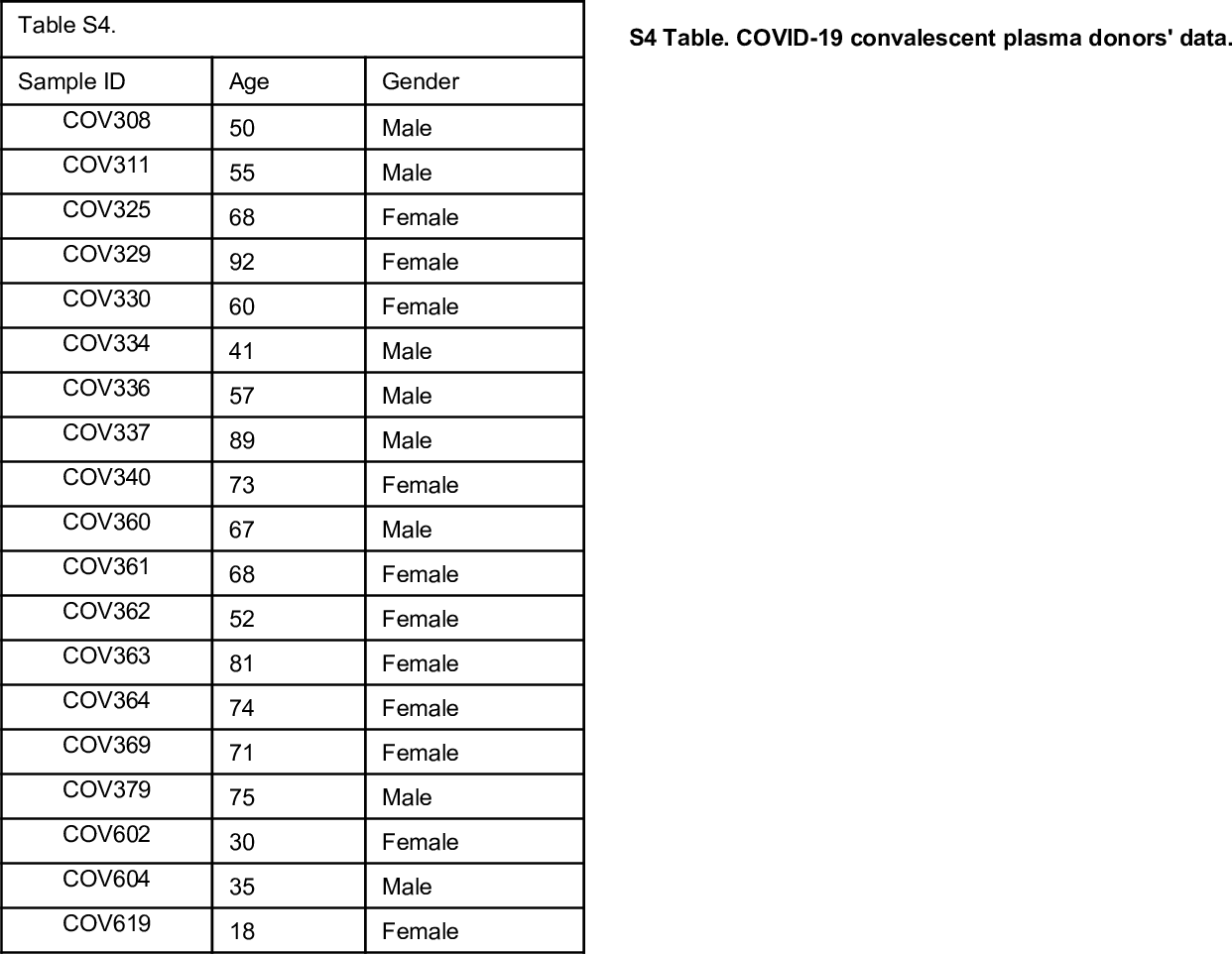

Supplement: S4 Table — (TIF) [file ppat.1010242.s011.tif]
