# Supplementary material for: SARS-CoV-2 variants with reduced infectivity and varied sensitivity to the BNT162b2 vaccine are developed during the course of infection
Source: PLoS Pathog. 2022 Jan 12;18(1):e1010242. doi: 10.1371/journal.ppat.1010242 (PMC8789181; doi:10.1371/journal.ppat.1010242)
Supplement: Attachment — Submitted filename: Point_by_point.pdf [file ppat.1010242.s012.pdf]

Comment:

Reviewer #1: Khateeb et al. used single genome sequencing (SGS) to characterize sequence variations in the SARS-CoV-2 spike (S) and nucleocapsid (N) genes from nasal swab samples. SARS-CoV-2 variants that successfully transmit from person to person have been the subject of intense investigation. The degree of variation that occurs within an infected individual is also of interest, however, because this may provide insights into virus evolution. The present study makes a good start towards addressing in vivo variation. However, the study has several limitations that reduce its significance. Among these, the authors do not conclusively demonstrate that the SGS approach yields an accurate measure of variant frequency. The number of individuals sampled is small and the conclusions that can be drawn from this study are quite limited. Finally, some technical concerns regarding the neutralization experiments were noted.

Response:

We thank the reviewer for acknowledging the importance of addressing SARS-CoV-2 intra-host variability and for pointing out the limitation of our study. We have modified the manuscript according to the reviewer's comments, which significantly improved the current version of the manuscript. We added two additional individuals to the study; we provided further information regarding the SGS analysis; we performed additional experiments in order to strengthen the conclusions of this study and improved the neutralization experiments.

Comment:

Reviewer #2: This manuscript aims to examine the intra-host variation of SARS-CoV-2 in the context of viral sensitivity or resistance to vaccine induced antibodies. By using single genome sequencing method, the authors uncover the sequences of low-frequency viral variants that exist in swabs of seven infected donors, and are less likely to be detected by regular RT-PCR reactions. Interestingly, based on S and N proteins sequencing many of the infected donors exhibited quasi-species in additional to the

“main” viral strain. As might be expected, many of the intra-host variants exhibited stop-codon mutations, making them nonfunctional, however a small number of variants had no apparent S or N- related defects, and of these some exhibited different levels of resistance to vaccine-induced antibodies.
As opposed to numerous other studies describing SARS-CoV-2 inter-host variation, where bulk viruses are sequenced, the topic of viral diversity within the same person is less addressed. Such analysis is interesting from both basic-virological and translational-medical aspects, and therefore this work is definitely worth publishing.
There are several typos and inconsistencies between the text and the figures that must be corrected before publication.

Response:

We thank the reviewer for recognizing the value of our study, its’ possible contribution to this research field and the possible impact of our analysis on translational SARS-CoV-2 studies. The reviewer raised important points that needed to be addressed. We have corrected our manuscript based on the reviewer comments.

Part II – Major Issues: Key Experiments Required for Acceptance
Please use this section to detail the key new experiments or modifications of existing experiments that should be absolutely required to validate study conclusions.

Comment:

Reviewer #1: 1. The samples prepared for SGS were amplified by nested PCR. Were control PCR reactions performed to determine at what frequency variation is introduced by the PCR? The authors indicate that they use a high-fidelity polymerase for the PCR, but it would be reassuring to directly test PCR mutation frequency.

Response:

We thank the reviewer for pointing out that despite the fact that we used high-fidelity Platinum Taq, control PCRs should be performed. In response to this comment, we first identified the maximum number of viruses that were isolated by SGS, which was 55 viruses. Then we single-genome amplified WT Wuhan-Hu-1 spike gene 55 times using an identical protocol that was used for analyzing SARS-CoV-2 intra-host variants. We demonstrated that following the single genome sequencing of the WT genomes, no mutations were found. This indicates that it is unlikely that the mutations that we have identified in the infected individual are a result of a PCR-mediated mutations.
Please see Supplementary Fig. S3 and text on page 6, third paragraph.

Comment:

2. From any individual swab, up to roughly 40 S or N genes were sequenced. Therefore, even variants that are detected only once may represent about 2.5% or more of the total population. It would be helpful for the authors to perform next generation sequencing on each of the swab samples and determine at what frequency the SGS variants are detected. This could establish how well the SGS approach quantifies variant frequency. It would also test whether variants are missed by the SGS approach.

Response:

We thank the reviewer for this important point. To address the reviewer comment we preformed bulk sequencing for five individuals from our study (we could not perform this analysis on all the individuals in our study due to the limited cDNA amounts). For this analysis bulk PCRs were preformed, and variant calling analysis was done to identify intra-host variants. Comparing these results to the SGS results revealed that bulk sequencing and variant calling analyses only identified variants that are frequent (more that 13%) and many of the variants that were identified by SGS were missed by the variant calling analysis. This is probably due to the exponential amplification of the high -frequency variant that dilutes the low-frequency clones. This is in accordance with what

we previously observed with HIV-1 samples isolated from infected individuals (unpublished data). However, this comparison also revealed one of the limitations of the SGS results, as this method failed to identify several variants that were identified in the variant calling analysis. Thus, we conclude that higher sampling size is required in order for the SGS results to accurately estimate the intra-host variant landscape.
Please see Supplementary Table S2, page 7 first paragraph, page 14 second paragraph and page 16 second paragraph.

Comment:

3. Figure 3 describes the results of the N gene sequencing. While the data are clearly described, it is unclear what conclusions can be drawn from these data, beyond the fact that variants can be identified. It would also be helpful to clarify why N was chosen for these experiments versus another gene(s).

Response:

The revised manuscript was modified based on this comment. First, we explain that we chose to focus on the N-gene since it is the most abundant viral protein in infected cells and is highly immunogenic. Please see page 7, third paragraph. In addition, since a recent report demonstrated that the pseudovirus system can be used to test the effect of N-protein mutations on viral particle assembly and infectivity (Abdullah M. Syed et al. *Science* 2021), we chose all N-protein variants with missense mutations that were identified in our SGS analysis and tested their effect on pseudovirus production and infectivity. Interestingly, we demonstrate that four of the tested N-protein mutations can significantly improve both pseudovirus production and infectivity, which is in contrast to the reduced infectivity seen with the spike variants that we tested.
Please see Figure 3C-D, page 8, second paragraph and page 13, first paragraph.

Comment:

4. Figure 5 assesses antibody responses in the individuals from whom swabs were taken for sequencing. The fact that individuals who harbor variants comprising a minor proportion of their viral load develop robust anti-spike antibody responses is not surprising.

Response:

Based on this reviewer comment we modified the text in order to better explain that the data in Figure 5 depicts the antibody levels and neutralization activity of new individuals who were recruited to this study and to whom SGS was not preformed.
Please see text on page 10, first paragraph.

Comment:

5. In Figure 5, does the strong naturalization by COV325 and COV329 reflect higher levels of anti-spike antibodies or is there some qualitative difference that makes these sera better? This could be clarified by performing the assay with the same amount of anti-spike antibody from each sample.

Response:

We thank the reviewer for raising this interesting question. We now address this in the text and explain that the strong neutralization seen in individuals COV325 and COV329 is likely because these two individuals had the highest titers of anti-spike IgGs.
Please see text on page 10, first paragraph.

Comment:

6. The data in Figure 6 lead the authors to conclude that the L1197I mutation within spike S2 significantly impacts neutralization efficiency. However, performing this assay again but using a finer dose response curve is needed. The authors report 50% neutralization titers (NT5). However, many of the curves have only one data point that lies between 100% and 0% neutralization. This makes determining an accurate NT50

problematic. 3-fold or 5-fold serial dilutions would give more precise data than the 10-fold serial dilutions that are used.

Response:

Based on this comment, we performed an additional neutralization assay experiment, in which we used three-fold plasma dilutions and also used new stocks of pseudoviruses that carry Wuhan-Hu-1 spike protein or the L1197I mutation and evaluated the NT_{50} values. The NT_{50} values observed in this experiment where lower than the values measured in Figure 6, since the viral titers of the newly generated pseudoviruses were lower. Nevertheless, in accordance with the data in Figure 6, the NT_{50} values required for neutralizing the L1197I mutant were significantly higher that the NT_{50} values that were observed when a WT pseudovirus was used.
Please see supplementary Figure S7 and text on page 11, first paragraph

Reviewer #2: none

Part III – Minor Issues: Editorial and Data Presentation Modifications
Please use this section for editorial suggestions as well as relatively minor modifications of existing data that would enhance clarity.

Comment:

Reviewer #1: 1. Figure 2. The units for the scale bar beneath the phylogenetic trees should be defined.

Response:

The units are now defined in the figure legends. Please see pages 20, second paragraph and page 21, first paragraph.

Comment:

2. Figure 3. The significance of the different colors in the pie charts should explained.

Response:

The figure was modified accordingly.
Please see Figure 3B.

Comment:

Reviewer #2: • I could not find in the manuscript how the viral load/titer was determined. Can the authors comment why the viral load/titer is different from Ct values for some samples in Fig 1B (for example for patients 4000 and 4065)?

Response:

We added an explanation in the methods section on how the viral load was calculated. There is a reverse correlation between the Ct values and the viral load (samples with high viral load will have a low Ct values). This is the reason that the Ct values and the viral load are different.
Please see page 15, first paragraph.

Comment:

• The authors did not address glycosylation issue – are some of the mutations they found affect addition or removal of glycan residues?

Response:

We thank the reviewer for drawing our attention to spike glycosylation sites. After examining the location of all the mutation that were isolated, we identified a variant that was isolated from two separate individuals (individual 4000 and individual 48559), which carry a mutation in the N-glycosylation site (C968T, T323I). An additional mutation in an O-glycosylation site was identified in individual 3953 (A1807G, N603D).

We discuss these findings on page 6 second paragraph, page 9 first paragraph and page 13, first paragraph.

Comment:

• Figure 2 is very hard to read and does not always consistent with the text. For example, in the text the authors write: “Further analysis of SARS-CoV-2 sequences from individual 3120 revealed S-gene variants with nonsense mutations (C1687T) in which a glutamine amino acid was replaced with a premature stop codon.“ however, looking at Figure 2, I was not able to find C1687T within the variants appearing in individual 3120, or any donor (I could find C1678T in donor 3953 – is this a typo?). Also, the authors write “Although the abundancy of each intra-host mutant was sparse, we also found a few S-gene mutations that were repeated in several viral isolates (nucleotide substitution A3300G and T1389C, Fig 2A).” Perhaps it is worth noting that the sequences repeated are all within the same donor 3807 and not shared between different donors as might be implied. The same is true for the bottom of page 6, the authors write: “S-gene variant C1686T that was isolated by our SGS approach was also found in 2x10-3 of the GISAID database” however, here too, I was unable to find variant C1686T in the Figure. The authors should make sure all the data in the Figure is consistent with the text. I also suggest that in Figure 2 the authors mark with different colors or shapes the variants/substitutions that exhibit nonsense mutations, as well at the common variants that appeared in more than one donor, or more than once in the same donor. The GISAID info can also be incorporated into Fig 2A to indicate which variant were [previously reported and which ones are new.

Response:

All typos were corrected in the text and in the figures: phylogenetic trees were redesigned, and more individuals (n=2) were added.

C1687T mutation was corrected in individual 3120 phylogenetic tree.

The repeated nucleotide substitutions A3300G and T1389C were repeated in the same individual 3807, and this is now noted in the text. Please see page 6, second paragraph and page 7 first paragraph.

C1686T was corrected and now appears in individual 3380 phylogenetic tree.

Figure 2 was edited according to the reviewer's suggestions: different colors were used for each substitution, and the undocumented mutations were marked. Please see Figure 2.

Comment:

• Why for the analysis of the N protein the authors analyzed only five donors and not all seven? Should be explained in the text

Response:

The reason that only 5 individuals were analyzed is that for few individuals we did not have enough RNA to perform the SGS to both the spike and the N protein. We have mentioned this in the revised manuscript.
Please see page 7, third paragraph.

Comment:

• Figure 3: I was wondering why the representation of S (Figure 2) and N different?

Response:

In the revised manuscript we modified Figure 3, to match the representation to Figure 2. Please see Figure 3.

Comment:

• Figure 3B – are the same colors (red) represent similar mutation (C168T) repeating in the different individuals? I suggest putting a legend for the different colors and their mutations in 3B. Mutations that repeat in more than one donor should be indicated clearly. Also, I suggest that the numbers of the substitutions to be indicated on top of the sequence alignment in 3A.

Response:

We modified the figure according to the reviewer's comments.
Please see Figure 3.

Comment:

• Similar Supplementary figure as the one made for S protein (Supplementary Figure 2) should be prepared for N protein to summarize all mutations.

Response:

Such a figure is included in the revised manuscript. Please see Supplementary Figure S2B.

Comment:

• More inconsistencies in the text – page 7 – the authors write that highest degree of variability was observed for donor 3120 who is 73 years old. However, in Figure 1 the clinical info states that this donor is 74 years old..

Response:

We apologize for this typo, which was corrected. The correct age is 74.
Please see page 8, second paragraph.

Comment:

• Donor 3120 exhibited high sequence diversity in both N and S genes, did the authors check whether the degree of S and N diversity correlate with each other? Do the diversities of S and N correlate with viral load?

Response:

There was no correlation between the diversity of the N gene and the S gene or the viral load. We discuss it on page 8, first paragraph.

Comment:

• The sentence in Page 7 “These variants were selected based on the mutations location and based on their absence or presence in the GISAID database “is not clear.

Response:

This sentence was modified. Please see page 9, first paragraph.

Comment:

• In Figure 4B it looks like all the S variants have lower p25 values and many of the variants have a dramatic 8-10-fold reduction in p24 levels compared to WT. Therefore, the S mutations could affect different stages of the viral production, such as packaging and protein synthesis. How can the authors be sure that the reduction in luminescence in 4D is due to reduced infectivity and not due to other mechanisms? Perhaps they should rephrase and use “reduced viability” instead?

Response:

We thank the reviewer for the suggestion that other mechanisms might mediate the reduce luminescence that was observed. We emphasize this in the discussion on page 13, third paragraph.

Comment:

• The authors probably submitted the manuscript before the emergence and sequence domination of the Delta VOC. Though the Delta VOC is mentioned once in the Introduction, I believe it will make the paper more relevant if the authors consider

updating both the Introduction and the Discussion sections accordingly so they mention more the Delta variant, and the resistance mutations it encompasses, while citing the appropriate references. Also, the more recent nomenclature of the VOCs should be added (i.e., B117 is named “Alpha”, B351 “Beta”, P1 “Gamma”, etc.).

<u>Response:</u>

We updated the introduction and discussion according to the reviewer’s comments. We also used the more recent nomenclature for the variants.
Please see page 3 second paragraph, page 4 third paragraph and page 14 second paragraph.
